# Exact research on the theory of the blackbody thermal radiation

**DOI:** 10.1038/srep37214

**Published:** 2016-11-23

**Authors:** Xin Yang, Bing Wei

**Affiliations:** 1 College of Physics and Optoelectronic Engineering, Xidian University, Shannxi, 710071, China; 2 Collaborative Innovation Center of Information Sensing and Understanding at Xidian University, Xi’an, 710071, China

## Abstract

After studying the normalized Planck equation in depth, a brand-new type of spectrum curves of blackbody thermal radiation is given. Two important parameters of the new type curves, namely relative width RW_η_ and symmetric factor RSF_η_, are defined. The paper points out that the experimental verification of the parameters has three significant applications: (1) Giving a method to measure temperature by detecting the radiation wavelength. (2) Determining the blackbody grade. (3) The temperature obtained from the law of the blackbody thermal radiation can be used as a criterion.

It is well known that, over the past hundred years, the theoretical basis of the blackbody thermal radiation is expressed in the form of Planck’s law, given by^1^,^2^,^3^,^4^


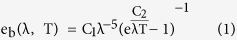


where C_1_ = 3.7415 × 10^−4^ W·μm^2^, C_2_ = 1.4388 × 10^−4^ μm^2^·K. **e**_b_(λ, T) is the monochromatic radiant emittance of the blackbody at the temperature T(K) and radiation wavelength λ(μm). For a given temperature, the **e**_b_(λ, T) and wavelength λ are related by the traditional spectrum curve of the blackbody thermal radiation^5^, as shown in Fig. 1. When the first derivative of function ***e***_*b*_(*λ*, *T*) with respect to λ is equal to 0, the relationship of the temperature with the peak wavelength λ_m_ of the curves in Fig. 1 results, which is the famous Wien displacement law^6^


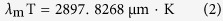


Under the development of the remote sensing^7^, night vision^8^, and thermal radiation thermometry technology^9^, many further studies on the blackbody thermal radiation have been carried out. For examples, by studying the inflection point feature along the both sides of the curves in Fig. 1, the lightwave equation of the inflection point was obtained as


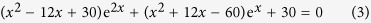


then the relationship of the wavelength at the left inflection point λ_li_ and right inflection point λ_ri_ with temperature T was derived from (3)^10^


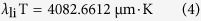



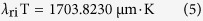


In order to find the wavelength domain of Eq. (1), ref. 11 proposed a normalized Planck’s equation


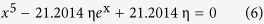


where the normalization coefficient η is defined by the following equation


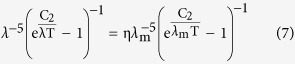


Obviously, η ranges from 0 to 1, inclusive. And the value of *x* in Eq. (6) is given by


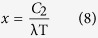


When η trends to zero, the variation of *x* or λ in Eq. (8) with temperature T was studied in reference 11. For η equal to 10^−6^, the conclusion of the reference is that the domain of the wavelength λ is between 88 and 1051(μm), when temperature between 200 and 6000(K).

Based on these studies above, the relationships between λ and η are investigated in this paper. And from the obtained relationships in this paper, a brand-new type of spectrum curves of the blackbody thermal radiation results, which is much clearer than that was. Then the relative width and symmetric factor of the spectrum curves are defined. Finally, a wavelength thermometry is presented, and the significant applications of the results obtained in this paper are shown as well.

## Results

### Normalized spectrum curve of the blackbody thermal radiation

**Figure 2:**
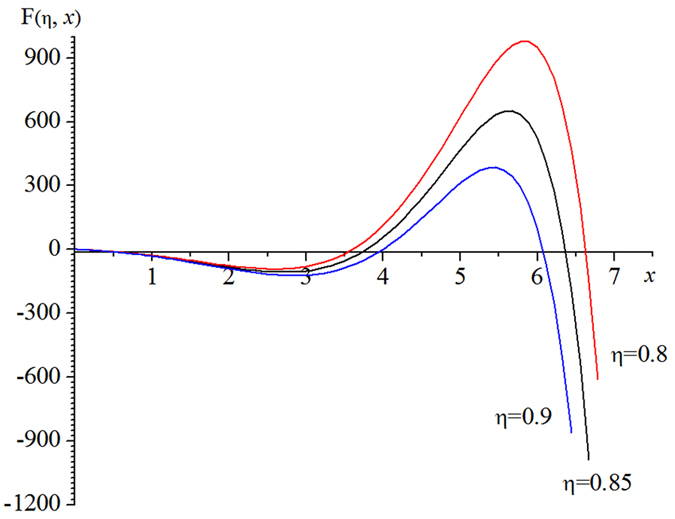
The curve of the left side of Eq. (6), when η = 0.8, 0.85, and 0.9.

For different values of η, Eq. (6) is a series of transcendental equations without analytical solutions, thus a brand-new normalized spectrum curve of the blackbody thermal radiation can be obtained from their numerical solutions. In order to numerically solve (6), it is necessary to determine its roots distribution. Programming to find out the roots of Eq. (6), Fig. 2 shows that, for a given η, the curve has two intersection points with the *x*-axis, which indicates that the transcendental Eq. (6) has two roots, denoted as *x*_ηs_ and *x*_ηl_ (*x*_ηl_ < *x*_ηs_). In accordance with Eq. (8), the two roots of *x*_ηs_ and *x*_ηl_ can lead to the wavelengths of *λ*_ηs_ and *λ*_ηl_, respectively, which locate on the left and right side of the spectrum curve with the corresponding value of η.

Programming to numerically solve (6), the relationship of η with its corresponding *x*_ηs_ and *x*_ηl_ results, as shown in Table 1. If necessary, any magnitude of *x*_ηs_ or *x*_ηl_ can be obtained by solving (6) based on the different value of η. RW_ηt_ and RSF_ηt_ in Table 1 are the theoretical results of the relative width and symmetric factor of the spectrum curves obtained from Eqs (14) and (15).

For η = 1, the values of *x*_ηl_ are equal to *x*_ηs_, which are both recorded as *x*_m_. Substituting *x*_ηs_, *x*_ηl_ and *x*_m_ of each η and the previous value of C_2_ into Eq. (8) can produce the following equations


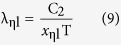



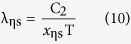



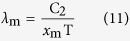


**Figure 3:**
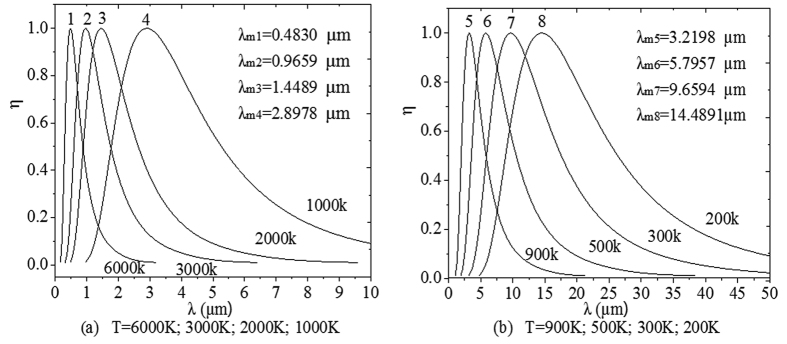
The brand-new type of normalized spectrum curve of the blackbody thermal radiation, where λ_mi_ represents the peak wavelength of each spectrum curve numbered i, i = 1, 2, …, and 8.

Then, Table 1 can lead to a brand-new type of spectrum curves of the blackbody thermal radiation, as shown in Fig. 3.

For a given Kelvin temperature T and under the condition of η equal to 1 in Table 1, the substitution of the *x*_m_ into (11) can lead to the same peak wavelength λ_m_ of Eq. (2). The short wave edge λ_ηs_ and long wave edge λ_ηl_, sitting on the both sides of the curve’s peak, can be obtained from Eqs. (9) and (10), then the relationship at this temperature of η with λ results. For example, when T = 1000 K, substituting any values of *x*_ηs_ and *x*_ηl_, which correspond to a certain value of η in Table 1, into Eqs (9) and (10) can obtain the values of λ_ηs_ and λ_ηl_ in Table 2. Then, using Table 2 can yield the η-λ curve 4 in Fig. 3(a). Similarly, other η-λ curves in Fig. 3 at different temperature can be obtained as well, which are the normalized spectrum curve of the blackbody thermal radiation presented in this paper. It should be noted that it will spend considerable software resources and time to solve Eq. (6), using the unknown of η in Table 1 to get a curve. Therefore, the data in Table 1 is already enough to be used in general conditions, and may also be used as a manual text to plot the other spectrum curves. In addition, the monochromatic radiant emittance of λ_ηs_ and λ_ηl_ at any temperature can be obtained from the right side of Eq. (7), when necessary.

### Feature description and application analysis on the spectrum curve

In order to accurately describe the characteristics of the normalized spectrum curve of the blackbody thermal radiation, the actually measured wavelength λ_ηs_, λ_m_ and λ_ηl_ can give a definition for the curve of the relative width RW_η_ and symmetric factor RSF_η_ as


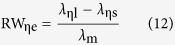



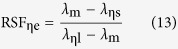


On the other hand, substituting the wavelength of Eqs (9) to (11), 10, 11 into (12) and (13) respectively, RW_η_ and RSF_η_ can be theoretically defined as follows


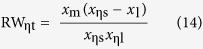



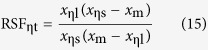


The RW_ηt_ and RSF_ηt_ of Table 1 are the theoretical results produced from (14) and (15), with the corresponding *x*_ηs_, *x*_ηl_, and *x*_m_. Obviously, RW_ηe_ and RSF_ηe_ are closely associated with η. But it is usually more concerned with RW_0.5e_ and RSF_0.5e_, i.e., η = 0.5.

For any of the RW_η_ or RSF_η_, the Eqs (12) and (13) are the experimental results, and Eqs (14) and (15) are the theoretical. Thus, if the experiment systems are reliable and the experimental results are consistent with the theoretical values, it will prove that the object tested in the experiment is a blackbody. In addition, the following equations can be derived from Eqs (9) to (11), 10, 11


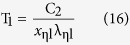



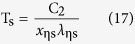



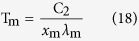


The Eqs (16) to (18), 17, 18 show that the true temperature of the tested object can be detected by measuring the wavelength of 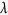
_ηs_, 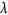
_ηl_, and λ_m_, namely, which provides a thermometry based on the measurement of the radiation wavelength.

## Discussion

Only the local spectrum characteristics of the blackbody thermal radiation have been studied in refs 6, 10, and 11 based on the traditional spectrum curve. In this paper, its global characteristics are studied based on the brand-new type of spectrum curves of blackbody thermal radiation as shown in Fig. 3. The results obtained in this paper have three important significances.

First, it provides a novel method to verify blackbody and its grade. From the Eqs (12) to (15), 13, 14, 15 given by the experimental and theoretical results, two errors of a and b can be defined as follows


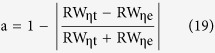



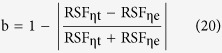


Then, using a and b can give an exact definition to the blackbody. When a and b are both equal to 1, it represents an ideal blackbody. When a and b are very close to 1, such as 0.9, 0.99, 0.999 and so on, it can define different grades of the actual blackbody.

Second, using an actual high-level blackbody as the experimental subject and applying a high-precision spectrometer to detect the radiation wavelength, it can be seen from Eqs (16) to (18), 17, 18 that, at a constant temperature, the actual temperature of a blackbody can be determined by measuring three wavelength values: the peak wavelength and two wavelengths corresponding to a suitable η. Therefore, the temperatures calculated from the three wavelengths have a cross-calibration function, which can fully verify the credibility of the measured temperature. As long as the errors between them are small enough, the temperature obtained from Eqs (16) to (18), 17, 18 can be used as a criterion.

Table 1, Fig. 3 and these two points above are the most important conclusions of this paper, which are enlightening and helpful for further researches on the blackbody thermal radiation. Unfortunately, there are no equipments for authors to do relevant experiments. So we can only provide the theoretical results here to share with readers.

Finally, based on the researches available on the blackbody thermal radiation, it must be noted for the earth’s thermal radiation that the microwave, which has been detected by the passive microwave remote sensing technology^12^, cannot be predicted by Planck’s law in Eq. (1), even at the nearly lowest temperature of the Earth’s surface^11^. To solve this problem for describing the characteristics of the gray body thermal radiation, authors believe, it maybe go one step further and deeply study Kirchhoff’s law^13^, and that will be our next research topic.

## Additional Information

**How to cite this article**: Yang, X. and Wei, B. Exact research on the theory of the blackbody thermal radiation. *Sci. Rep.* **6**, 37214; doi: 10.1038/srep37214 (2016).

**Publisher's note:** Springer Nature remains neutral with regard to jurisdictional claims in published maps and institutional affiliations.

## Figures and Tables

**Figure 1:**
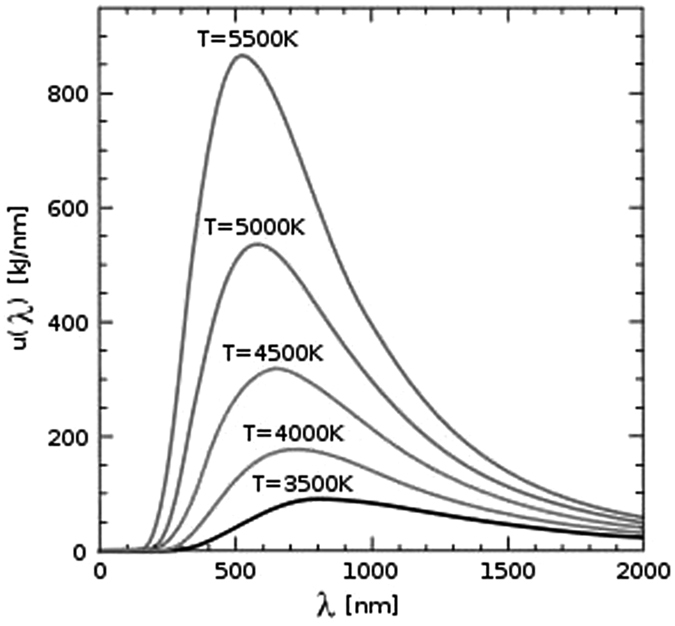
The traditional spectrum curve of the blackbody radiation.

**Table 1:** Values of *x*_ηl_ and *x*_ηs_ obtained from Eq. (6) with different η.

η	x	0.0100	0.0500	0.1000	0.2000	0.3000	0.4000
*x*_η_	*x*_ηs_	15.1368	12.6168	11.4295	10.1358	9.3001	8.6505
	*x*_ηl_	0.7496	1.1958	1.4862	1.8818	2.1916	2.4682
RW_ηt_		6.2952	3.7584	2.9060	2.1484	1.7314	1.4375
RSF_ηt_		0.1195	0.1924	0.2417	0.3114	0.3685	0.4213
0.5000		0.6000	0.7000	0.8000	0.8500	0.9000	1.0000
8.0966		7.5942	7.1131	6.6236	6.3615	6.0722	4.9646
2.7326		2.9986	3.2796	3.5960	3.7795	3.9946	4.9646
1.2036		1.0019	0.8158	0.6311	0.5331	0.4252	0.0000
0.4736		0.5281	0.5879	0.6581	0.7003	0.7512	1.0000

**Table 2:** Data of the normalization coefficient η with wavelength λ when T=1000 K.

λ(μm)	η	0.0100	0.0500	0.1000	0.2000	0.3000	0.4000
λ_η_	λ_ηs_	0.9505	1.1404	1.2589	1.4195	1.5471	1.6633
	λ_ηl_	19.1949	12.0326	9.6808	7.6458	6.5649	6.8294
0.5000		0.6000	0.7000	0.8000	0.8500	0.9000	1.0000
1.7770		1.8946	2.0227	2.1722	2.2617	2.3695	2.8981
2.7325		4.7983	4.3871	4.0011	3.8069	3.6018	2.8981
